# Star-Tracker Algorithm for Smartphones and Commercial Micro-Drones

**DOI:** 10.3390/s20041106

**Published:** 2020-02-18

**Authors:** Revital Marbel, Boaz Ben-Moshe, Roi Yozevitch

**Affiliations:** 1 Department of Computer Science, Ariel University, Ariel 4070000, Israel; revi85@gmail.com; 2 Department of Electrical Engineering, Ariel University, Ariel 4070000, Israel; yozevitch@gmail.com

**Keywords:** star tracker algorithm, global orientation sensor, accurate orientation for autonomous robotics

## Abstract

This paper presents a star-tracking algorithm to determine the accurate global orientation of autonomous platforms such as nano satellites, UAVs, and micro-drones using commercial-off-the-shelf (COTS) mobile devices such as smartphones. Such star-tracking is especially challenging because it is based on existing cameras which capture a partial view of the sky and should work continuously and autonomously. The novelty of the proposed framework lies both in the computational efficiency and the ability of the star-tracker algorithm to cope with noisy measurements and outliers using affordable COTS mobile platforms. The presented algorithm was implemented and tested on several popular platforms including: Android mobile devices, commercial-micro drones, and Raspberry Pi. The expected accuracy of the reported orientation is [0.1°,0.5°].

## 1. Introduction

Since the dawn of history, man has had to navigate in space. The navigation problem can be essentially divided into two different sub-problems: where am I and where am I heading. The answer to the latter used to be based upon the starry sky. In fact, star navigation was the main and foremost tool for both marine and land navigation for centuries. If you know the stars, you will never get lost, said the famous proverb. Nowadays, technology provides unbelievable accurate solutions to the ancient navigation problem, both in terms of localization and orientation. Contemporary GNSS receivers report an absolute position and heading with an average error of a few meters and less than 1°. Alas, as technology progresses, so do the demands. GNSS suffers from inherent accuracy errors, especially in urban regions [1]. Moreover, the accuracy levels reached by inertial measurements units (a smart fusion of accelerometer, gyroscope, and magnetometer) are insufficient for many contemporary applications.

For example, commercial magnetometers have an angular resolution of ≈1° and are extremely sensitive to magnetic noise. Thus, they cannot be implemented in tiny drones due to motor interference. A gyroscope’s drift and an accelerometer’s noise are part of their inherent nature and cannot be fully overcome even when fused with other sensors [2].

This work addressed the heading problem by offering a fusion of an ancient concept with modern technology—A star-tracking algorithm implemented on a mobile device.

A mobile star tracker can determine a vehicle’s exact orientation with accuracy levels unreached by IMUs and GNSS.

Moreover, motivated by recent GNSS spoofing threats [3,4,5,6], the use of star trackers on unmanned aerial vehicle (UAV) drones, aircraft, autonomous vehicles, and vessels may help detect such attacks and increase the robustness and accuracy of autonomous navigation systems. Star tracking may also improve the accuracy results of mobile mapping, whose process requires accurate global orientation in every measurement [7].

In a nutshell, a star-tracking device is comprised of a camera equipped with a tracking algorithm. The basic principle behind any star-tracking device is that earth-orbit satellites (and, by extension, earth-located viewers) perceive stars as fixed stationary points with known distances. The algorithm seeks to estimate the camera-viewing direction using a star image obtained from the camera. star trackers are more accurate and reliable and allow for attitude estimation without any prior information [8,9]. While originally invented for earth orbiting satellites, we believe that even earth-located applications can benefit greatly from star-tracking devices. Thus, this work focuses on the general framework and algorithms for star-tracking using COTS devices (smart-phones).

The star-tracking algorithm is divided into three consecutive steps:

1. Detect star centers with subpixel accuracy,
2. Assign a unique catalog identification (ID) or false tag to each star and
3. Calculate the camera-viewing direction.

The system input is a noisy image or images of the night sky. The first step focuses on extraction of the star coordinates (subpixel resolution) from the frame(s) [9]. The second step is the core of the algorithm, naming the stars by their unique ID. The ID can be obtained from lists such as the Hipparcos catalog provided by NASA. This step’s output is a list of all visible stars with their names (string) and positions (x,y coordinates within the frame). However, how is this achieved?

In order to track the “real” stars from the image, the algorithm must perform an image registration between the captured image and the known star positions obtained from the catalog. Most star tracking algorithms make use of the fact that the angular distance between two stars, as seen from an earth located observer, remains almost fixed.

**Figure 1:**
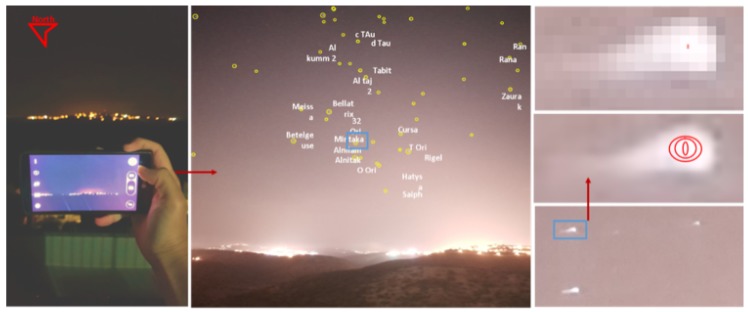
The star tracking algorithm on Mobile smart phone. **Left**: step 1: capture an image of the night sky via smartphone. **Right**: step 2: the image processing algorithm identify the stars pixels centroid in the image in super resolution. **Middle**: star identification phase—naming the stars according to the star catalog. Lastly, calculate the Image angle from the north-orientation (red arrow). Note that the image was taken while holding the phone with the relatively long exposure leading to blur effect as shown on the right.

Note that the Brute Force algorithm (presented in Section 2.2) is time consuming because there are over 30,000 stars in the catalog. This can be improved by using a grid-based search. Figure 1 demonstrates the three phases of the orientation calculation of a smart- phone camera using a picture of the night sky and the star-tracking algorithm.

### 1.1. Related Works

Acquiring absolute orientation is a great necessity for UAV and other autonomous vehicles for both navigating and mapping. Geometric vision based methods are commonly used to compute the accurate precise exterior orientation parameter (EOPs) from an image. The traditional approach is to use photos taken of the ground, find image features points and match them to the ground control images. This is due to the fact that image features’ extraction algorithms are very efficient and run relatively fast (canny edge detection, for example). In the man-made environment, for example, many algorithms use 3D lines as features for ground control points’ registrations and for building the image orientation modeling; meaning determines the image orientation [10,11].

This work aims to acquire an absolute orientation via star tracking obtained from mobile devices. There are indeed a few mobile star-mapping applications available on the Android-Play-Store and Apple-Store (Sky Map, SkyView, Star Chart, etc.); however, these apps utilize the GPS and IMU of the mobile device in order to simulate a map of the stars of the night sky resulting in poor accuracy. The proposed algorithm estimates the device self-orientation from the stars as obtained from the camera and correlated with the star-database.

There is a vast difference between a satellite star tracker and an earth located mobile star tracker (as elaborated in the next section). However, the stars registration phase is basically the same and this work builds on previous works and algorithms conducted in this field. Most of the algorithms for star trackers were designed for space applications such as image-satellite camera aiming. However, some research has used star tracker hardware for ground based rover navigation application e.g., [12].

First-generation star trackers were able to detect a few bright stars and deliver some focal-plane coordinates to the spacecraft computer [13]. In most cases, calculating the spacecraft’s attitude in space had to be done at the ground station. The second generation of star trackers used powerful space-qualified microcomputers to perform the attitude calculation on-board. Modern star trackers calculate space coordinates of the stars and transmit them to the device directly [14]. In other words, modern star trackers calculate their self-space coordinates in real time [14]. At the core of the star-tracking algorithm is the ability to identify star patterns and match them to the star catalog. There are numerous strategies to identify star patterns. One common technique, called the Grid Algorithm, was presented by Padgett and Delgado [15]. This technique places the stars on a predefined grid; the grid density determines the output resolution. The technique helps to overcome the disadvantage of the conventional angle-matching method. Unfortunately, one disadvantage of this technique is its expensive runtime.

An improved version of the grid algorithm uses a chosen star from the field of view (FOV) as a pivot star (center). The other star coordinates are translated according to their distances from the pivot and oriented to align with the axis created by the pivot and its closest star [16].

The Search-Less Algorithm suggested by Mortari [17] sought to improve the identification runtime using his k-vector search solution. The algorithm searches for k pairs of matching stars instead of searching the full catalog. A k-vector table is comprised of all catalogued star pairs that could fit in a predefined FOV over the whole sky. A slightly more sophisticated version of the search-less algorithm is the Pyramid Method. This method uses a preconstructed table of all catalog star pairs that can appear on a frame (given the camera FOV and a magnitude threshold). It then searches for a four stars pattern (called a pyramid) using an O(1) search technique called the k-vector search (also developed by Mortari [18]). This algorithm reduces the runtime dedicated to identifying stars to $O(k^{2})$ where *k* is the number of possible matches in the database for the star pyramid. However, this algorithm assumes the image has been accurately calibrated, which is unlikely when using mobile COTS cameras [19]. In 2009, Lang et al. developed a geometric hashing technique to solve the inability to identify stars in night sky frames without calibration. Their technique requires no first guess or scaling. In this method, a database of geometric ratios of small group stars in the catalog is created. The database is a multidimensional binary tree (kd-tree), in order to allow an efficient search [20]. Recently, the Tetra calibration-less Star Identification algorithm was suggested by Brown and Stubis [21]. Their algorithm uses minimum complexity and database access in its catalog search by using a hash table database. It outperformed earlier methods for star identification in runtime and FOV distortion. However, this algorithm also assumes narrow FOV (up to 10°) in order to get accurate results, which is not the case in mobile phones. Lastly, Yuji et al. [22] suggested the “Nearest Neighbor Star Search Approach.” This method relies on the fact that each star has connections with some adjacent stars. Hence, by using the star catalog (on continuous mode), unknown stars can be identified by tracking the nearest star from the previously recognized star. For a comprehensive survey on star-tracking algorithms, see [23].

### 1.2. Our Contribution

This work presents an algorithm for star tracking suitable for implementation on land based mobile devices. To the best of our knowledge, this is the first work to present such star-tracking implementation on COTS mobile devices. While there are other novel star tracking algorithms [24,25,26], they were not designed to work in atmospheric environment but in space on satellites.

This work focuses on designing a robust star-tracking algorithm that can effectively handle outliers such as optical distortion created by atmosphere conditions and partial, nonline-of-sight and star-like lights (e.g., airplanes and antennas). The suggested new algorithm can handle a wide range of star patterns and has an efficient expected runtime, which makes it applicable for low-end, mobile-embedded platforms with limited computing power (e.g., mobile phones). The initial implementation of the algorithm has an expected accuracy of [0.1°,0.5°], which is significantly more accurate than existing micro-electro-mechanical systems (MEMS)-IMU solutions and can be embedded in mobile platforms such as drones, autonomous cars, vessels, and nano-satellites. Finally, based on simulations and lab experiments, the expected accuracy of the presented algorithm on well calibrated COTS phones can reach a sub-pixel resolution of ≈ 0.01°.

## 2. Preliminaries

This section elaborates on several necessary definitions and properties regarding the star-tracking algorithm. The section starts with listing the challenges in an Earth-Located star tracker based on COTS camera (in opposition to space dedicated star-tracker sensors). Then, technical observations and definitions regarding the star-tracking model are presented. Finally, a naive brute-force approach and its limitations is discussed.

### 2.1. Earth-Located Star-Tracking Algorithm: Challenges

Traditional star trackers were designed and built to function in outer space. Thus, many earth-related issues were neglected. The most important earth-related issues are:

- Wide FOV. As opposed to space-tracking devices that use narrow FOV (15° × 20°) [27]), earth-designed algorithms should be able to handle a wide camera FOV because most accessible mobile devices have standard large FOV sensors (60°–80°). Furthermore, due to atmospheric interference, earth-located cameras capture a significantly lower number of stars. Therefore, a wide FOV is necessary to increase the number of captured stars.
- Fast Tracking. Although some second-generation star trackers perform LIS searches on each frame [28], the majority of star-tracking algorithms are coarsely divided into two consecutive phases: star acquisition (registration) and star tracking. Space-located algorithms can allow the first phase to be relatively slow. On the other hand, earth-located algorithms do not have this time luxury because the first registration phase should be calculated as fast as possible. This problem is commonly referred to as the Lost-in-Space (LIS) problem.
- Efficiency. A real-time algorithm cannot afford the wasteful inspection of an entire star database. The proposed algorithm should provide an efficient way to extract saved data from a gigantic database.
- Confidence Analysis. The algorithm should define and provide a confidence figure. Smoothing algorithms (e.g., Kalman filter) can also benefit from weighed results.

As stated above, similar to any GNSS operation, the orientation process is comprised of two consecutive phases: the acquisition phase (LIS) and the tracking phase. The acquisition phase assumes no prior knowledge. We begin our discussion with the acquisition problem, presenting a set of observations and definitions, followed by a brute force star-tracking algorithm.

#### 2.1.1. Observations

The algorithm was developed under the following observations:

- Registration is the process of identifying pixels in the image as real stars. Full registration cannot be accomplished without identifying exactly two stars. When three or more stars are identified, the system becomes over-determined and a modified RMS equation can be used:
  (1) $$RMS=\sqrt{\frac{\sum_{i,j=1}^{n}(dist\left(s_{i,j}\right)-dist\left(p_{i,j}\right){)}^{2}}{n}}$$
  where $dist(s_{i,j})$ is the angular distance between two stars (i,j) in the database as described in the next subsectoin and $dist(s_{i,j})$ is the Euclidean distance between two stars (i,j) in the frame (pixels).
- Although identification of two stars is sufficient for full registration, the proposed algorithm seeks to match star triplets due to the relatively large number of outliers (as elaborated in Section 2.1).
- The angular distance between two stars as observed from earth can be addressed as fixed. Therefore, a fixed angular star database can be used.

#### 2.1.2. Definitions

- Camera Frame *F*: represents a 2D pixel matrix from which a set of *observed stars* can be computed. Each frame star p∈F has the following attributes: 2D coordinates, intensity, and radius.
- Star Database: Several star databases are available online. One of the most well known (and the one used in this paper) is the Yale Bright Stars Catalog (BSC) [29]. Every star s∈BSC has the following attributes: name, magnitude, and polar orientation.
- Angular Distance between two stars: The distance between two stars as seen from Earth. Each star in the BSC has the spherical coordinates: right acceleration and declination. The angular separation between two stars $s_{1},s_{2}$ is computed using this formula:
  (2) $$AD\left(s_{1},s_{2}\right)=sin\left(dec_{1}\right)sin\left(dec_{2}\right)+cos\left(dec_{1}\right)cos\left(dec_{2}\right)cos\left(RA_{1}-RA_{2}\right)$$
  The angular distance units are degrees.
- Star Pixel Distance: The distance between two stars in the frame. Because the stars in the frame are represented by x,y coordinates (pixels), the distance is computed using the L2 formula. The star pixel distance unit is pixels.
- Distance Matching: There is a need to define a transformation formula T(p)=s,p∈F,s∈BSC that will allow us to match the frame stars’ distance (pixels) to the angular distance (angles). For two catalog stars $s_{1},s_{2}$ and camera scaling: *S* the stars’ pixel distance will be:
  (3) $$StarPixelDistance\left(p_{1},p_{2}\right)=S*AD\left(p_{1},p_{2}\right)$$
- Star Labeling: Given an image *F* as set of pixels $<p_{1},\ldots ,p_{n}>\in F$ and a stars database BSC. A star label is set according to its matching star *s* in the BSC. We define for each star pixel p∈F: A label L(p) that is its matching stars s∈SC. If a match was not found, we will label the star pixel *F* (false).

### 2.2. Brute Force (BF) Algorithm

The brute force (BF) algorithm seeks to find a match between the stars captured in each frame and the a priori star database. A common practice is to take three stars from the frame and match this triplet to the stars catalog. Algorithm 1 describes this naive method.
**Algorithm 1:** Stars identification BF algorithm.
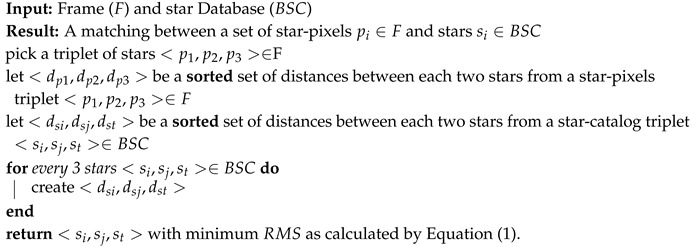


**Orientation:** After labeling each star in the frame, the orientation is calculated in the following manner: Given a match: $S:=<s_{1},\ldots s_{n}>\in BSC$ with $P:=<p_{1},\ldots p_{n}>\in F$, the algorithm will return a matrix $O_{\left[1,3\right]}$ and a vector $T_{2}$ so that S=O×M×P with *M* being the rotation matrix in order 3 and *T* being the translation vector (from pixel (0,0)). This matrix defines the device orientation.

Given an image frame *F* and its global orientation *T*, the validity of *T* with respect to *F* can be computed as follows: for each star in the image (s∈F), compute the global orientation of *s* denoted as $T_{s}$ and, for each $T_{s}$, compute the distance between *s* and the closest star in the database. The overall weighted sum (e.g., RMS) of these distances can be treated as the quality of the global orientation *T* with respect to *F* and the star database [27].

The algorithm’s complexity is $O(N^{3})$, with |N| being the size of the star database, which is ≈10,000. This number **only** represents the brightest stars in the night sky. Naive algorithms tend to work well in perfect scenarios. However, the proposed brute force algorithm does not address the following real-world issues:

1. **Measurements Inaccuracy:** The captured images hold inherent distortion errors. Section 3.1 will elaborate on those errors. However, an accuracy parameter should be defined. This accuracy figure is determined from the camera resolution, the lens FOV, and weather conditions.
2. **Full Stars Pixel Labeling:** The naive algorithm can only identify three stars’ pixels on the frame. However, there is a need to label these star pixels as either “match” or “outlier” because identifying outliers is a main task of this algorithm (as discussed in the Motivation subsection). The labeling challenge in our framework is divided into two main issues:
  **False positive/negative errors.** Earth-located frames contain two kinds of inherent errors. The first is false-positive objects that appear in the sky as stars (e.g., airplanes). In this paper, we treat those errors as outliers. The second is false-negative stars that cannot be captured in the frame (e.g., blocked by clouds or buildings). A flexible algorithm should cope well with a considerable amount of outliers (up to 25%).
  **Confidence.** The Star Identification algorithm cannot be systematic because the data it receives are not always accurate. Hence, there is a need to assign a confidence parameter to each match. The confidence parameter will be applied for each star pixel. We define $\forall R\in F,R=<p_{i},L\left(p_{i}\right)>$ and a confidence parameter C(R) to be the pixel star and label confidence. This number will represent the probability of the star label being true. Section 3.2 will describe an algorithm that uses probabilistic methods to identify outliers and compute confidence parameters for each star pixel match.
3. **Runtime Efficiency:** The brute force algorithm time complexity is $O(N^{3})$. This is not a time efficient algorithm. The registration phase must be computed much faster. Section 3.1 will discuss a solution for this constraint.

## 3. The Star-Tracking Algorithm

**Figure 2:**
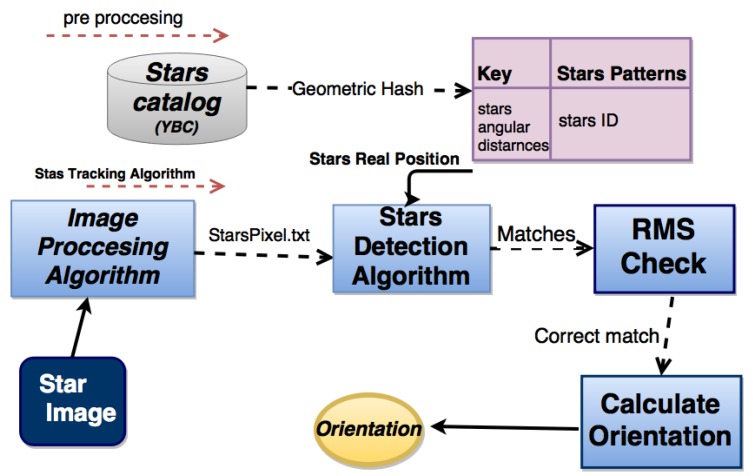
The main components of the Star-Tracking algorithm.

A mandatory phase of every star-tracking process is identifying the stars on a given image. Through identification, one is able to find the optimal matching (or registration) between each image star and the corresponding star in the database. Figure 2 shows the building blocks of the star tracker.

Algorithm 2 has three parts: The preprocessing phase, using a Stars Pattern Hash Table (SPHT)), the real-time processing and a validation algorithm. The preprocessing algorithm is a unique hashing technique that reduces the real-time search but also allows the algorithm to overcome measurement inaccuracies. The real-time part of the algorithm extracts the star pixels from the frame (into a text file that contains the star coordinates) using image processing. Then, the algorithm labels each star–pixel in the frame by pulling the star’s data from the hash map and grading each star in the frame accordingly. Finally, the validation algorithm grades and verifies the orientation correctness and accuracy.

### 3.1. Preprocessing Algorithm: SPHT

To avoid a massive amount of searches (brute force), the algorithm uses hash map techniques that, when one fabricates its own database, have a runtime search complexity of O(1). The question is, therefore, a hash map of what? Single star coordinates? The algorithm computes each star’s triangle distances in the database and saves each triangle as a unique key in this table. The value of each key will be the names of the stars as they appear in the BSC.

The hashing process goal is to a priori construct interesting star triplets (with their respective angles). For efficiency considerations, this is done by dividing the sky with a virtual grid. From this, one can compute all the visible star triplets. A key factor in the robustness of the method is to utilize overlay cells in the computation. The hashing algorithm runs only once to create the system database. Thus, the $O(N^{3})$ hashing complexity does not affect the general algorithm complexity, which, like any hash map, has O(1). Each star triplet creates a unique set of three angular distances. For each triplet of stars in the catalog, we save a vector of catalog star numbers as the key and a vector of sorted angular distances between the stars as the value (the triangle implementation can be extended to n patterns of stars). We define:

- Starset: a set of three stars in the catalog, denoted as $<s_{1},s_{2},s_{3}>\in BSC$.
- Key function of a starset: $key\left(<s_{1},s_{2},s_{3}>\right)=sort({⋃}_{i,j=1}^{3}Round(distance(\left(s_{i}-s_{j})))\right)$.
- Value function of a starset: $value\left(<s_{1},s_{2},s_{3}>\right)={⋃}_{i=1}^{3}s_{i}.name$.
- SPHT: SPHT = ⋃ all Starsets in BSC that might be detected by the star tracker camera (with magnitude less than some fixed value, often taken as 5.) and their distances from each other are less than the camera aperture.

#### 3.1.1. Measurement Error and Accuracy Figure

Because measurement errors exist (due to lens distortions, etc.), it is difficult to match computed triangles to a priori known triangles in the database. Therefore, a rounding method should be applied. Seemingly, a naive rounding would be sufficient. However, the inaccuracies (distance distortions) do not exceed the sub-angle (degrees) range. For example, we would like to distinguish occasionally two pairs of stars an with angular distance gap of 0.1°, but pairs with an angular distance gap of 0.01° should be considered identical. This subsection presents the AL parameter analysis, defined as follows:

- The AL parameter is a number between 0 and 1. This parameter is predefined and will dictate the way we save the star triplets in the hash map.
- The AL parameter is a rounding value that multiplies each distance in the BSC before creating a key.
- A well defined AL will determine the algorithm probability to pull star sets from the map and identify them. The lower this parameter is set, the lesser the probability that each key is unique. On the other hand, a low AL value will increase the probability of retrieving a key with a distorted value.

**Figure 3:**
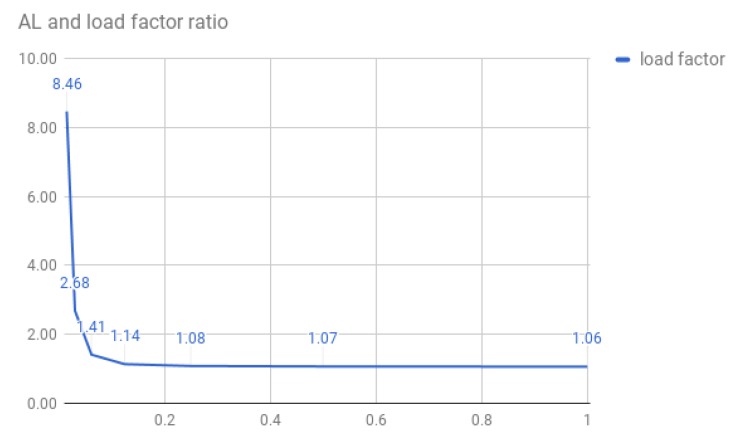
Accuracy level and star detection ratio. The *x*-axis represents the AL parameter on a logarithmic scale. The *y*-axis represents the ratio between all keys in the database and the candidates’ patterns (load factor).

In all, the higher the accuracy, the greater the chance of missing similar patterns in the image frame. We consider this to be a good trade-off. Before determining the best AL to use, we performed some tests on the BSC to simulate different scenarios. **The First Test** was done by counting the values for each key under various ALs. We used the BSC database to create the hash table. The graph depicted in Figure 3 represents the probability of finding a star pattern of size 3 corresponding to the AL. For example, for an AL figure down to 0.2 (load factor of ≈1), there is only a single candidate for each triplet; however, for an AL of 0.065, the load factor is ≈8.5.

The AL parameter should be set after considering the system constraints. Denote “LoadFactor” to be the ratio between the total number of triangles in the hash table and the number of unique keys. For example, if a large distortion due to bad weather, wide angle lens distortion (in particular wide FOV lens), inaccurate calibration, or atmospheric distortion is expected, then we would set the AL to be lower than 0.2. However, if we expect almost no distortion, then we can set the AL higher and reduce the algorithm runtime.

In the **Second Test**, we measured the effect the distorted distances have on the AL parameters. We also simulated different distance errors for every two stars in the BSC with different ALs. The distance errors were set by using Gauss’s probability distribution model.

For each two stars $s_{i},s_{j},s_{t}$, expected deviation err and AL, we created a key:

(4) $$\begin{array}{c} key_{e}\left(s_{i},s_{j},s_{t}\right)=sort\left(\overset{k}{\underset{i,j=1}{⋃}}Round\left(distance\left(\left(s_{i}-s_{j}\right)+randGaus\left(err\right)*AL\right)\right)\right) \end{array}$$

where randGaus(err) is a random value with Gauss normal distribution with mean value of 0 and standard deviation is the error expected. We also created a real key:

(5) $$\begin{array}{c} key_{r}\left(s_{i},s_{j},s_{t}\right)=sort(\overset{k}{\underset{i,j=1}{⋃}}Round(distance(\left(s_{i}-s_{j}))*AL\right) \end{array}$$

**Figure 4:**
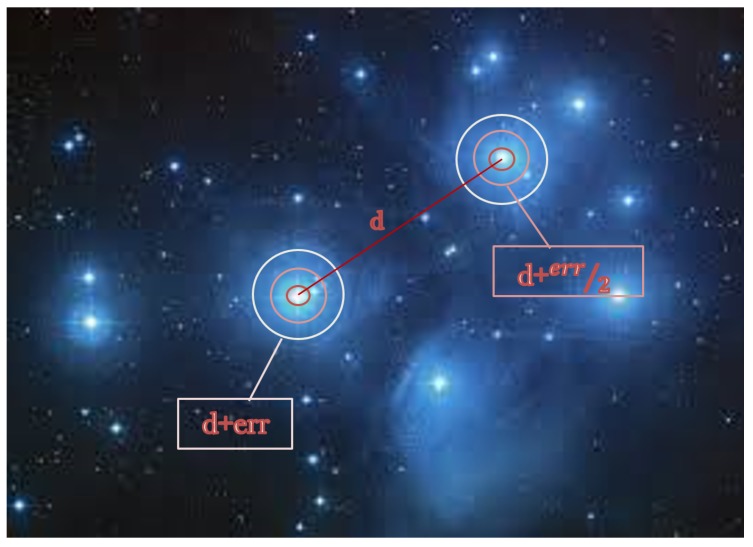
A graphic representation of Gauss’s distribution of standard distance deviation err around the stars.

Figure 4 shows the graphic sense of the err distribution we defined.

**Figure 5:**
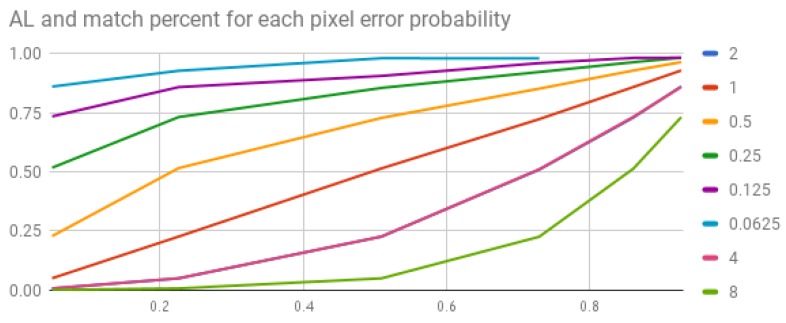
Accuracy level and distribution effect on right matching probability. The lines represent different AL values in logarithmic scale. The *y*-axis represents the probabilistic error we added to each distance. The *x*-axis represents the probability to retrieve star triplets from the BSC with that error.

The next graph, Figure 5, shows the effect different distortion and AL values have on the probability to retrieve the correct keys from the hash table:

According to these results, what is the AL that should be commonly used in earth-located scenarios? The results show that, to obtain an over 0.8 probability, which is achieved by retrieving the right value from the BSC with error probability of 0.5, the AL can be close to 1°. However, in case the error probability is 8°, we will have to set the AL to 0.015625 to get 0.7 probability in order to retrieve the correct value. Why is this graph important? Bear in mind that the essence of this work is adaptating the star-tracking algorithm to distorted sky images obtained from an earth-located COTS smartphone.

Empirical experiments in Section 4.1 hypothesized the distance gaps can be up to 1.5° due to camera lens distortion and atmospheric effect alone. Therefore, the AL parameter has to be set to a maximal value of ≈0.1 in uncalibrated cameras.

Hence, an improved star-identification algorithm should handle a case of multiple results for each key due to the large impact factor required to handle large distortions.

#### 3.1.2. Camera Calibration Effect

When discussing a distance gap, there is a need to consider the camera calibration effect. Camera calibration should reduce the gap to a minimum by transforming the image pixels to “real world” coordinates. Two factors affect frame distortion: radial distortion, caused by light rays bending nearer to the edges of a lens than the optical center tangential distortion, and a result of the lens and the image plane not being parallel. In our case, we considered only the radial distortion because the stars are so far that their angles to the camera are almost parallel.

**Figure 6:**
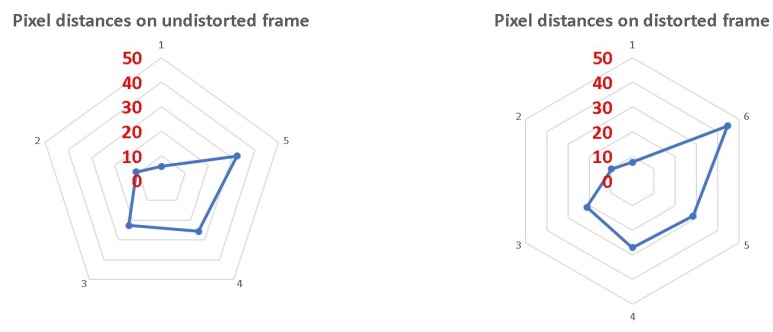
Distorted and undistorted frame distance deviations. Each pentagon in the graph represents a 10-pixel deviation from the real distance in the frame.

Because we used mobile device cameras with wide FOV, the radial distortion on the frame edges can cause distortion in distance calculations. For this reason, we expected more accurate results after camera calibration. This means that distances of the stars in the frame should be calculated according to the camera calibration parameters (focal length, center, and three radial distortion parameters) after the frame is undistorted. However, experiments showed that even calibrated frames have a distance deviation of $\frac{1}{2}$°. We tested the distance in eight frames of two stars taken from different angles before and after we undistorted the frames using a calibration parameter. We used a Matlab calibration framework to calibrate and undistort the frames. In Figure 6, we see the calibration reduce the distance gap. However, the gap still exists. These results emphasize the need for an algorithm that can overcome the distance deviation. We can also rely on the fact that we know the real angular distance between two stars (from the BSC) and use it to calibrate the scene after first identifying and obtaining more accurate orientation.

Due to the distance gap and the need to reduce calculations in real time, we propose a hash map model of star triplets with the addition of a rounding parameter. This model should help real-time orientation calculation.

### 3.2. The Real-Time Algorithm (RTA)

To minimize search time on the BSC while improving efficiency, we used hashing as described in the previous section, Preprocessing Technique. In this subsection, we describe an algorithm that searches star patterns of size 3. However, the algorithm can be expanded to work any star pattern of size >2. This algorithm will run after the construction of SPHT. The setConfidence(p,v) algorithm is elaborated in the next subsection.

Algorithm 2 assigns each star triplet in the frame with its matching star triplet from the catalog. For each match, the algorithm also sets the confidence parameter (number between 0 and 1).
**Algorithm 2:** Stars identification improved algorithm
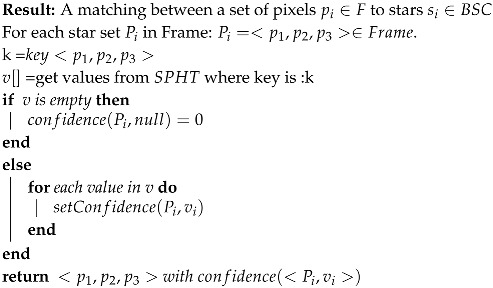


#### Multiple Marches Using Pattern Intersections and Confidence

As discussed in Section 3.1.1, there may be times when we want to utilize low AL in SPHT construction; for example, in bad weather, we should expect the distances to be inquorate. In such cases, we can obtain more than one set of stars from our hash table, and the algorithm will have to decide which set is the true match. The algorithm we present here suggests a solution for when the SPHT algorithm returns more than one set of matches. Note that:

- The algorithm will run where for each pixel triangle: $<p_{1},p_{2},p_{3}>$ of stars on the frame there are two or more matches $<m_{1},\ldots m_{n}>$, where $m_{i}$ is three stars from the catalog $<s_{i},s_{j},s_{k}>$ so that: $L\left(<p_{i},p_{j},p_{k}>\right)=<s_{i},s_{j},s_{k}>\in BSC$.
  In other words,

For every star $p_{i}$, there are some matches $<m_{i},\ldots m_{j}>$.

We define:

- A table SM that will hold the possible matches $L(p_{i})$ (labels) for each star in the $p_{i}$ frame (retrieved from SPHT).
- Confidence confidence(p,L(p) parameter will be added to each star pixel and labeled $<p_{i},s_{i}>$ in the frame.

This part of algorithm will determine the best pixel in the frame to use for tracking. The algorithm will return a star and its label with the highest confidence. Another way to think of this is to search the stars that appear in the highest number of triangle intersections. The confidence parameter range and its lower threshold will be discussed in the Results section. The algorithm will get an array of stars pixel triangles and their possible labels $ISM={⋃}_{0}^{n}<p_{i,j,k},L\left(p_{1}\right)\ldots L\left(p_{k}\right)>$ as input. This algorithm is the implementation of the setConfidence method described in the previous algorithm.

**Figure 7:**
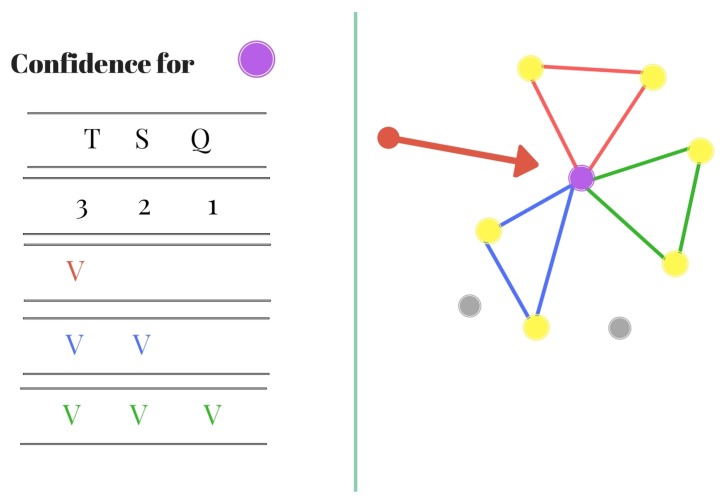
Calculating the match confidence for triplets with several keys.

**Figure 8:**
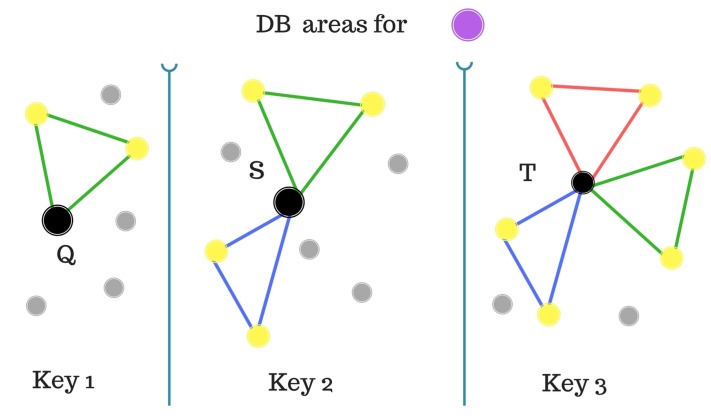
Database areas for stars patterns.

Figure 7 and Figure 8 visually explain the confidence calculation process.

Figure 7 demonstrates the SM table of possible labels for the stars (according to the different locations in the catalog) and the confidence for each label. Figure 8 shows the purple star’s possible locations in the catalog according to its closest stars.

The star-labeling algorithm can also be utilized to track celestial objects. This implementation is for airplanes or telescope-satellite tracking. In case such implementation is required, we can set the algorithm to track the outliers instead of the stars because they are already labeled.

### 3.3. Validating and Improving the Reported Orientation

The RTA reports the most suitable identification for each star in the frame and the confidence of this match. However, due to significant inaccuracies and low AL values, there is a need to validate the reported orientation. Moreover, the accuracy of the orientation can be improved using an additional fine-tuning process incorporating all available stars in the frame. The validation stage can be seen as a variant of a Ransac method [30] in which the main registration algorithm identifies two or more stars. According to those stars, the frame is being transformed to the BSC coordinates. Then, each star in the transformed frame is being tested for its closest neighbor (in the BSC). The overall RMS over those distances represents the expected error estimation. The validation Algorithm 3 performs the following two operations: (i) validates the reported orientation and (ii) improves the accuracy of the RTA orientation result. In order to have a valid orientation ($T_{0}$), at least two stars from the frame need to be matched to corresponding stars from the BSC. The validation process of $T_{0}$ is performed as follows:
**Algorithm 3:** Validation Algorithm for the Reported Orientation**Result**: The orientation error estimation **Input**: $S,T_{0}$: *S* a set of all the stars in current frame. $T_{0}$ the reported RTA orientation.
Let $S_{T_{0}}=S^{\prime }$ be the stars from *S* transformed by $T_{0}$. For each star $s^{\prime }\in S^{\prime }$
**search for its nearest neighbor**
$b^{\prime }\in BSC$, let $L<s^{\prime },b^{\prime }>$ be the set of all such pairs. Perform a filter over *L* - removing pairs that are too far apart (according to the expected angular error). ErrorEstimation = the **weighted RMS** over the 3D distances between pairs in *L*. **return**
ErrorEstimation

To implement the above algorithm, the following functionalists should be defined:

- **Nearest Neighbor Search**. This method can be implemented using a 3DVoronoi diagram [31], where the third dimension is the intensity/magnitude of the star.
- **Weighted RMS**. The weight of each pair can be defined according to the confidence of each star in the frame.

The weighted RMS value is then used as an error-estimation validation value. In case we have a conflict between two or more possible orientations, the one with the minimal error estimation will be reported.

Finally, the validated orientation can be further improved using a gradient descent in which the estimation error should be minimized.(for an implementation of such a minimizing RMS method between 3D point clouds, see the CloudCompare [32] open source).

## 4. Experimental Results

This section presents both simulation and experimental results of the SPHT algorithm. Furthermore, because the distance gap (deviation) has a large effect on the algorithm precision, an error modeling is presented as well. Finally, we discuss a few technical issues regarding actually implementing the suggested algorithm on mobile COTS devices such as Android mobile phones. We begin with the distance gap modeling experiment.

### 4.1. Distance Gap Experiment

To examine the distance gap, we picked 40 frames from the Orion star constellation for testing. The frames were taken at different locations in different countries and weather conditions. As explained above, those conditions have been known to affect the angular distance of two stars as seen from earth. The purpose of this test was to learn how large this distortion would be and to implement it on our algorithm. The experiment was split into two parts. In each part, we checked the difference between the distances of every two stars on several frames:

- Part 1. Testing star images taken in the same time and place to see the effect light distortion has on the tracking algorithm. Only one frame was used as base data for the following frames on the video.
- Part 2. Testing star images taken at different times and places to help adjust the LIS algorithm mode for first detection.

The frames analysis was conducted in four steps:

- Star image processing. We extracted each of the stars’ center pixel into a 2D array $F_{1},F_{2}$= $<s_{1},\ldots ,s_{n}>,<t_{1},\ldots t_{n}>$ of size *N*,
- Manually matched the stars from each frame $F_{1},F_{2}$,
- Calculated the pixel distances for each pair $s_{i},t_{i}\in F_{1,2}$ in every frame to two distances sorted as array $D_{1}$ and $D_{2}$ and
- Calculated and returned the maximum gap of each pair distance for all i $G_{i}=d_{i}\in D_{1}-d_{i}\in D_{2}$.

These tests confirmed our presumption that the distance gap between two frames can be quite large. We noticed a gap as big as 1.5° between frames from different locations.

**Figure 9:**
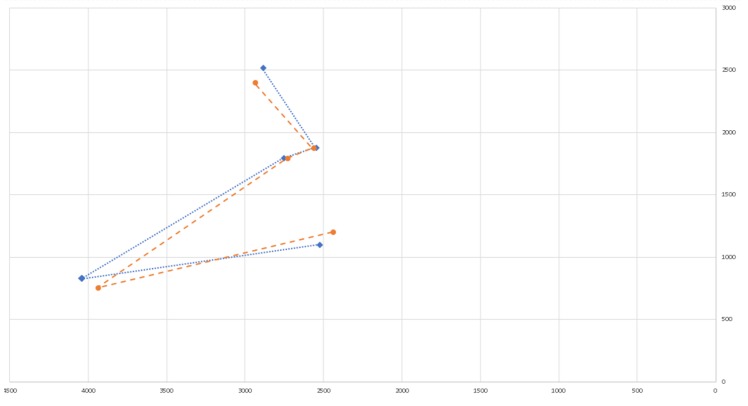
Two frames of stars from different locations.

Figure 9 provides an example of two frames of the Orion constellation from different locations. The red dots represent the stars’ centers from one frame and the blue dots, from another frame. This demonstrates that the gap between two similar stars (in circle) can be very large.

**Figure 10:**
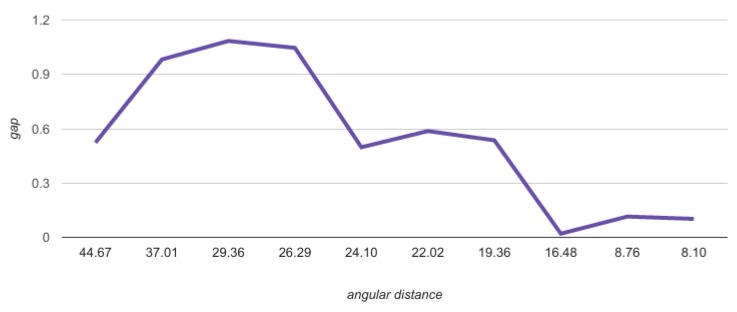
Angular distance gap between two stars in the ratio of its size.

In addition, we saw that the gap increased with distance. The next Figure 10 shows the result of the distance gap experiments on our frames.

Figure 10 shows that the larger the distance between the stars (in two frames), the larger the gap can be. It also shows that, for close stars, the gap can be under 0.3 degrees.

### 4.2. Simulation Results

To test the algorithm’s correctness, a simulation system was created. The system fabricates a synthetic image from a set of random coordinates from the database using the orthogonal projection formula via a Stellarium system [33]. In order to simulate real-world scenarios, the system also fabricates “noisy” frames by shifting some star pixels and adding some outlier stars. The output image is a close approximation of the night sky as seen from Earth. The $\frac{pix}{deg}$ ratio of the simulated frames was 74.922, similar to most COTS cameras on which the algorithm will be implemented.

The first part of the simulation tested the first mode of the tracking algorithm (LIS mode). The tests focused on the following five parameters in particular:

- The minimum AL needed for identification,
- Confidence testing, dealing with outliers and false-positive stars in the frame, and
- The algorithm’s runtime in each scenario.

#### 4.2.1. Accuracy Level Parameter

In the last subsection, we showed that the distance gap can be very large. However, a small gap between the stars’ distances also exists (under 0.26°) for more than ≈10% of the stars in the frames. There are several reasons for this gap besides the atmospheric effect because the frame was taken manually and with no camera adjustments. Naturally, we also saw that the distance gap is affected by its length.

The simulation’s results show the minimum AL needed to get the keys from the database is 0.01. This means that the average number of triangles for each key is 3 (see Section 3.1.1).

Hence, we tested the algorithm with an AL of 0.01 on several simulated frames with possible gaps of 0.2°–1.5° to see the percent average of matching stars. The algorithm was able to correctly label an average of 25% of the stars. We stretched the algorithm boundaries by adding up to 0.25% outliers to the frame, so as to not affect the results. Interestingly, the number of **real** stars on the frame influenced the algorithm’s success. That is, the algorithm was able to identify and match more stars because there were more real stars on the frame.

Whereas an AL of 0.01 on this algorithm has a probability of 0.25 to set a star label, the right labels had high confidence in our results. This observation is highly important to the algorithm’s LIS mode. This is because the LIS algorithm requires only two stars to determine primary orientation. Note that, after the first identification, we can also elevate the AL parameter because two close frames should have a small gap and give more accurate results. The confidence parameter of each of star is a value represented by the amount of triangles retrieved from the database that contains the star match. In conclusion, the accuracy level required for earth-located star tracking is 0.01. We simulated the SPHT algorithm on several simulated stars frames with artificial gap (0°–1.5°) and discovered that the algorithm was able to set the right label for 25% of the stars. Although not optimal, this result can be useful especially in the LIS mode because the confidence of the results was very high. In the following subsection, we analyze this parameter for all tracking scenarios.

#### 4.2.2. Confidence Testing

The algorithm’s confidence parameter plays a crucial part in deciding whether one or more of the stars on the frame was correctly identified. We added this parameter to the algorithm to solve the problem of retrieving many keys for each star triplet in case of low AL. In this case, one needs to consider the triplet’s “neighbors” in the frame and let them “vote” for each star label. The label that receives the highest confidence value is most likely to be true. However, it is not inevitable that the highest confidence match is wrong. In this subsection, we show how to distinguish correct from incorrect labeling as well as the low threshold for each AL.

Analyzing the calculated confidence for each star in the frame reveals that this parameter is heavily dependent not only on the AL parameter of the algorithm, but also on the number of keys the algorithm managed to retrieve from the database. Because the confidence of a star also depends on the number of “real” stars in the frame, we computed it as the ratio between the number of possible triangles that point to this label and the stars in the frame.

The Star Confidence parameter is defined by Algorithm 4, but we normalized this parameter to depend on the AL and the amount of triangles retrieved from the SPH algorithm in the following manner: For each star,

(6) $$s_{i}\in F,Confidence\left(s_{i}\right)=\frac{T}{Num}$$

with *T* being the number of all the triangles retrieved from the SPHT for this frame, and *F* and Num are the number of stars in that frame. As explained in the previous section, true labels should have more than one triplet. Therefore, we expected the right matches in the frame to have a confidence of 2 or more. We experimented with this theory on our simulated frames, testing frames with between 15 to 25 stars and frames that contained outliers.

**Algorithm 4:** Best match confidence algorithm

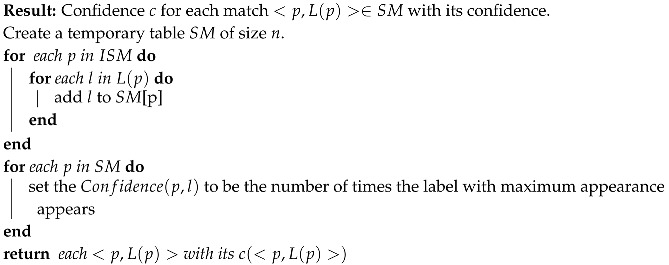



**Figure 11:**
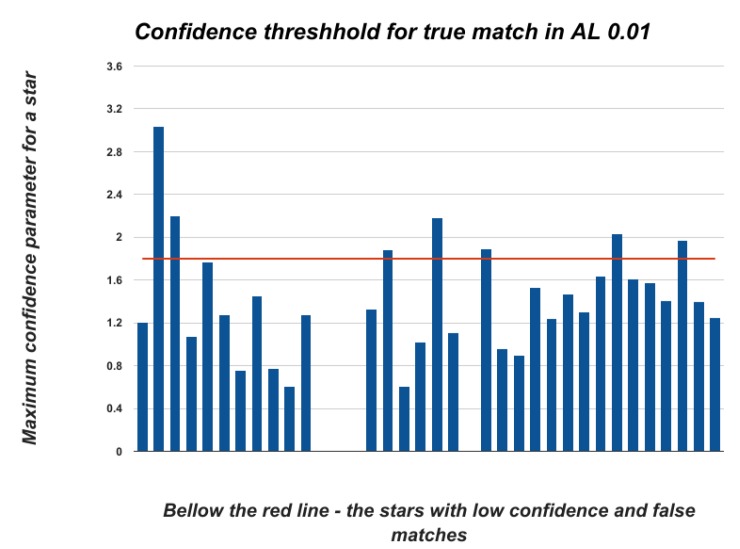
Confident thresh-hold for true match in AL 0.01.

**Figure 12:**
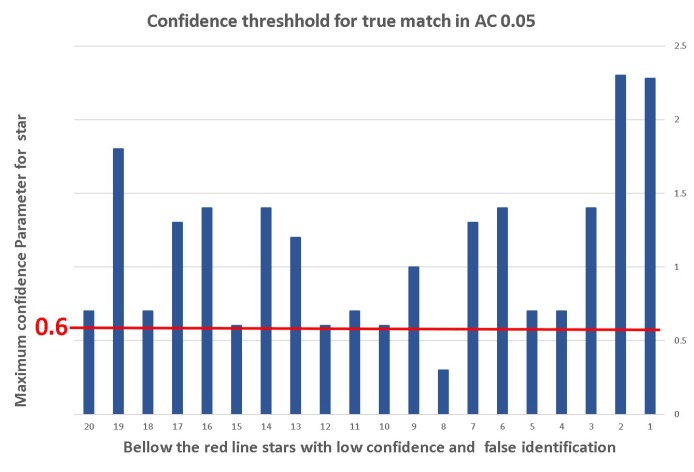
Confident thresh-hold for true match in AC 0.05.

Figure 11 shows that the confidence parameter threshhold for a correct match in a frame with 20% outliers and 0.2° gap is ≈1.8. This means that, if the SPHT algorithm identified a star match with confidence 1.8 or more, then this match is true and we can calculate the frame orientation based on this result. In higher ALs, we obtained a lower threshold for true matching. Figure 12 shows that the confidence parameter threshold for the correct match in a similar frame is under 1.

#### 4.2.3. Simulation Runtime

The algorithm was able to compute a complete identification for the synthetic images. The average runtime in the case of clean stars was ≈1.2 millis per image.

Another observation was that frames with 10% or less outliers have lower thresholds. However, because we cannot predict the number of outliers the frame has, we cannot relay on a low threshold in the LIS scenario.

Table 1 presents the algorithm’s runtime (in milliseconds) on different AL and various numbers of stars in the frame. The average runtime is not influenced by the algorithm results, meaning that the ability of the algorithm to identify the stars does not depend on the time this algorithm requires to run. The algorithm will require more time when the AL parameter is low (under 0.05) because the algorithm search predicted more triangles in case the AL was low.

### 4.3. Field Experiments

**Figure 13:**
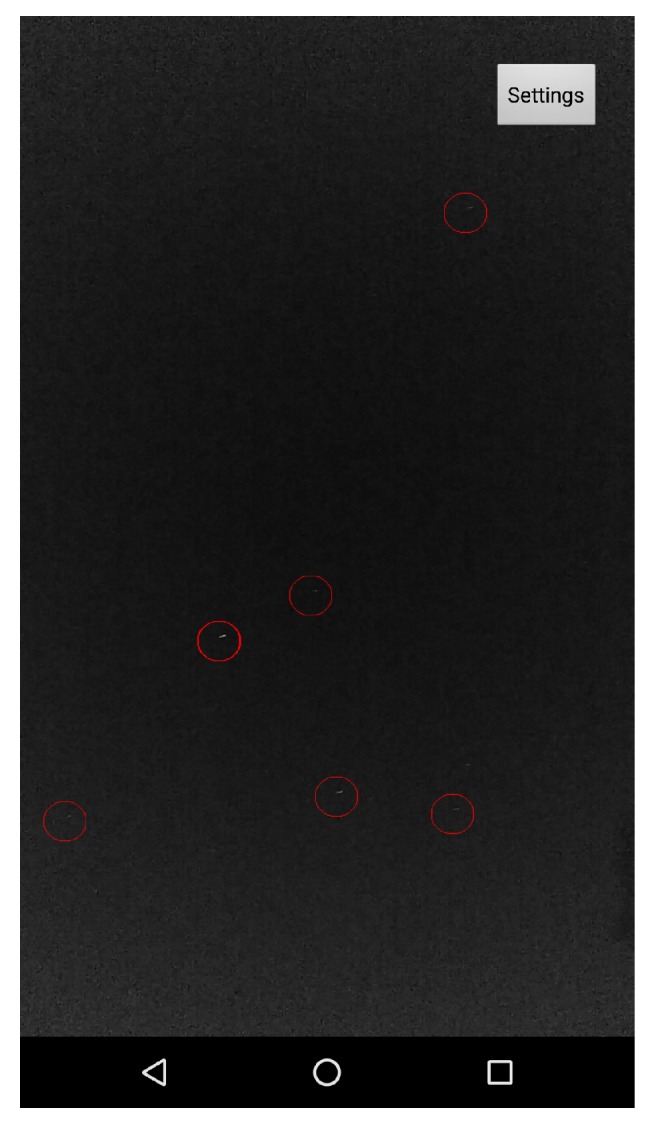
Android star-detection application: The stars are surrounded by red circles (“Big Bear” constellation). This image was captured using a Nexus 5x Android device. The stars are detected in real time (5–10 Hz) in 1080 p (FHD) video resolution. Note that this image contains significant light pollution at the lower side of the image.

To evaluate the performance of the proposed algorithm, we conducted a set of field experiments. A preliminary version of the suggested algorithm was implemented as an Android app and tested on several Android smartphones. The main goal was to show that the proposed algorithm can work in real time on COTS devices which are not stationary (all images were captured while the phones were held in hand). At the first stage of the experiment, we simply tried to be able to capture stars. Figure 13 shows the ability of an Android device to detect stars in real time (5–10 fps video in 1080 p resolution, full high definition; FHD).

**Figure 14:**
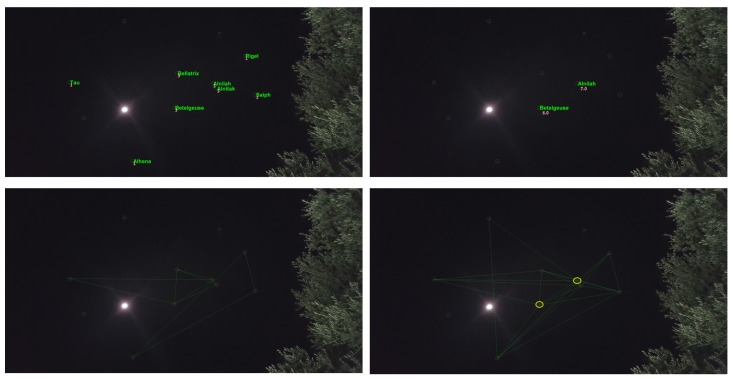
The upper frames represent star frames with their correct identification and match grade (confidence). The lower frames represent the triangles pulled from the hash table using the SPHT algorithm. The difference in the results is because the algorithm was set to different AL each time. The right figures represent the algorithm results when AL was set to higher numbers. The left figures represent the algorithm when AL was set to lower numbers.

After having the ability to capture stars in real time, the application converts the star image to a list of star pixels. This list is fed to the algorithm that computes the star registration, as shown in Figure 14 in which the registration was performed with respect to the BSC star data set. The star catalog (BSC) gives the stars’ positions, like any celestial object, in an equatorial coordinate system, meaning right acceleration and declination. Therefore, we first had to convert the stars’ angular distances as they appeared in the catalog to the pixels in the frame before the hashing process; we did so using the transformation described in the preliminaries [33].

Figure 14 shows the algorithm labeling process. The number near each match represents the confidence that the algorithm has attached to this match. Only matches with relatively high confidence are actually true matches. The confidence threshold for a correct match in this case was 2.75, and the AL parameter was relatively low because we expected light pollution to cause measurement inaccuracies.

**Figure 15:**
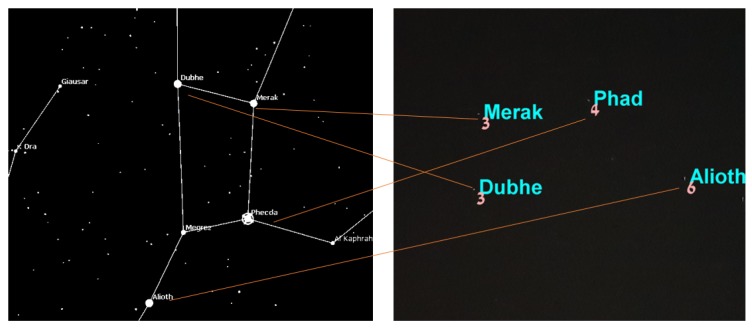
The algorithm implementation on Samsung’s Galaxy S7,S8,S9 smartphones. The right figure is the frame of the big bear constellation with star identification. The the left figure is the DB simulation of the big bear constellation as presented in the stellarium tool.

Finally, there is a need to discuss the accuracy of the results given possible inaccuracies in image processing. In this experiment, we used a simple super resolution to find each star-pixel center (in subpixels). Note that the average angular size of a star in the frame was [0.1°,0.2°]. Figure 15 depicts the typical way stars appear on the frame. The image process will allow us to improve the orientation accuracy about a single pixel ($\frac{1}{50}$ degrees). Finally, the SPHT algorithm can improve the accuracy to a subpixel AL (lower than $\frac{1}{100}$ degrees) using multiple star matching and through time. The calculation of the star pixel can be done with great accuracy using one of the star-centroid algorithms [34]. Such algorithms can improve the star center to subpixel accuracy, which was shown to be as accurate as 0.002 degrees [34]. We were unable to reach such accuracy in our preliminary smartphone implementation, so we conclude that the suggested algorithm was able to reach a pixel-level ($\frac{1}{50}$ degrees) true orientation accuracy on calibrated smartphones.

### 4.4. Global Orientation—Accuracy Testing

In this subsection, we present a methodology for testing the accuracy level of the suggested star-tracking algorithm. In general, the problem of computing the orientation using a star tracker can be defined as follows: Given an image of stars (*F*) and an aiming cross (a given point *x* in the *F*), compute the global orientation towards *x* (the frame cross). In order to test the accuracy of the global orientation reported by the suggested star tracking algorithm, we use the following notion of ground-truth (GT) test: Given an image (*F*) of *n* stars, let $F_{k}$ be the set of *k* stars which were identified in *F* (by the star tracking algorithm). For each $s_{i}\in F_{k}$, we can now perform a ground-truth accuracy test using the following: (i) Remove $s_{i}$ from *F* (denoted as $F^{\prime }$); (ii) Run the star tracking algorithm on $F^{\prime }$; and (iii) Compute the global expected position of $s_{i}$ in $F_{k}^{\prime }$ using a gradient descent over the star-catalog angular distance from $s_{i}$ towards all the other stars in $F_{k}^{\prime }$ (This process is basically estimating the frame position of $s_{i}$ which minimizes the RMS angular errors over the database location of the stars in $F_{k}^{\prime }$. Finally, compare the location of $s_{i}$ in the frame *F* to the estimated one and report the angular distance as the accuracy-error.

**Figure 16:**
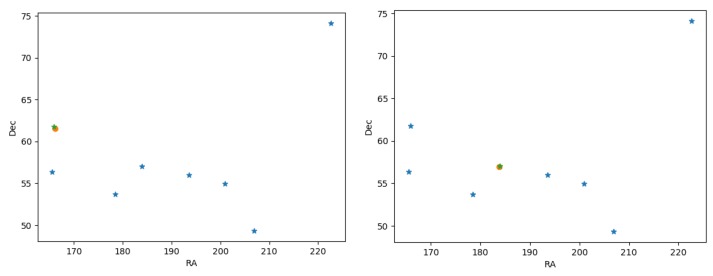
Orientation error example: the “Big bear” stars constellation in Ra,Dec coordinates (angular). Left: a 0.3° error-accuracy, the estimates orientation is marked by an orange circle, in green - the real image-based pixel of the tested star (“Dubhe”). Right: a 0.1° error-accuracy, the estimates orientation is marked by an orange circle, in green, the real image-based pixel of the tested star (“δ UMa”). The difference in the orientation accuracy is mainly due to the location of the tested star in the image. Stars within the 30° FOV tends to have a sub 0.1° error angular rate.

Figure 16 presents two examples of 0.3° and 0.1° global accuracy error rate.

Observe that, using the above gradient descent method, the global orientation to any “pixel” (*x*) in the frame can be computed. The frame angular distance from *x* to each identified star in $F_{k}$ will be computed, and then the global orientation of *x* will be calculated using gradient descent over the Ra,Dec location of *x*. Denote that the RMS value at *x* can be addressed as the expected accuracy of the global orientation of *x*.

### 4.5. Implementation Remarks

In this subsection, we cover technical aspects required to implement the suggested star-tracker algorithm on mobile COTS devices such as Android smartphones.

The first major implementation challenge regarding the construction of a COTS star tracker is to find a platform capable of shooting stars. Intuitively, we would like to use a platform capable of detecting stars at least as well as a human does. This research started in early 2015, when no phone we tested was able to properly shoot stars under average conditions. The Google Nexus 5× was the first device we were able to use to detect stars in real time (see Figure 13). Later, we found that Samsung’s Galaxy S6 produces better star images.

A preliminary set of experiments tested the algorithm ability for fast tracking by comparing two real night-sky images. One image was used as a dataset reference, and the other as taken by the camera. The frames were taken from different locations at different times and under different weather conditions. We chose the Samsung’s Galaxy S6 camera to take these photos with no calibration or modification. Thus, we expected the distance gaps to be between 0.5° and 1.5°. These preliminary experiments showed that the angular star-distance errors might get larger than 1.5°, but, in most cases, one degree was a common expected error for uncalibrated smartphones. In such cases, an AL of (0.01) was shown to be a more proper value for the AL parameter. The expected accuracy of the reported orientation is highly correlated with the quality of the camera calibration, which can be a somewhat complicated process in practice. We found the BoofCV [35] software to be the most flexible and advanced tool for calibrating Android cameras.

Lately, we found that the Android smartphones such as Samsung’s Galaxy S7,S8,S9 (and above) are great candidates for star tracking. Figure 14 demonstrates the ability of the Galaxy S7 to capture high quality star images, even in the relatively suboptimal conditions of city light pollution, full moon, and local lights. Note that the images in Figure 14 were taken using auto mode while holding the phone in hand. Based on the ever-improving quality of phone cameras, we argue that most mid-range smart-phones manufactured after 2017 are suitable for star tracking in terms of image quality.

**Figure 17:**
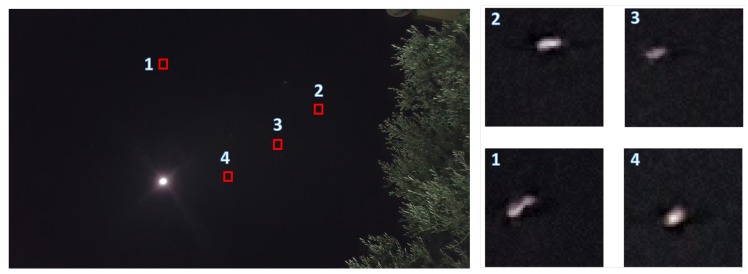
Star Pixels excepted inaccuracy. The four rectangles on the left are enlarged parts of the frame on the left. Each of the rectangles size is 50×50 pixels and in it are examples for star appearance on the frame.

Recent smart-phones allow better low light sensitivity, which implies an improved star detection capabilities in the night sky. One example of such mobile device is the “Samsung Galaxy S9”. Figure 17 represents the algorithm result on star-images as captured by this device. Since the lens of this device is relatively calibrated and therefore the frame is less distorted, we were able to detect and identify most of the stars in the frame with AL of 0.05 and more.

### 4.6. Star Frames Data Set

One of the goals of this work was to provide an empirical basis for research on star detection and tracking. To this end, we have collected hand-taken star frames from three types of smart-phones, a Raspberry Pi camera, and images from a time-lapse camera; see [36].

## 5. Discussion

In recent years, significant performance improvement has been made in off-the-shelf cameras. This progress was driven by the mass market of ever-improving cameras for the smart-phones and drones industries. Moreover, the huge production volumes lowered their price to a ridiculous minimum. However, such cameras are optimized for the mass market and hence cannot be modified.

The algorithm presented in this paper utilizes the inherit capabilities of those contemporary cameras. In the context of star-tracking, the four most relevant attributes of those COTS cameras are:

1. Wide FOV lenses,
2. Highly sensitive low light sensors,
3. Built-in low distortion lens,
4. Computing efficiency.

**Wide FOV lenses:** The wide FOV is required in a land-based star detection algorithm. Earth light-pollution decreases the probability to detect stars in urban regions (unlike the optimal conditions above the atmosphere). Wide lens cameras capture a large part of the sky in each frame and thus increase the probability of finding stars. Space designed star-trackers mostly consider narrow FOV; therefore, they are not suitable for land-based applications.

**Highly sensitive low light sensors:** Although low light sensors weren’t implemented in those COTS cameras for star tracking purposes, they are beneficial in the star tracking process, especially in cloudy environments. This feature saves the need for long exposure in most cases and allows for running the suggested algorithm on dynamic scenarios. **Built-in low distortion lens:** The camera calibration process to eliminate distortion is relatively simple yet allows only a modest accuracy improvement. In our experiments, we discover that most mobile cameras produce images with acceptable low distortion (they might be factory-calibrated), performing additional camera-calibration has a minor significance on the overall accuracy level.

**Computing efficiency:** The fact that image processing can be done using a wide range of programming languages and on many platforms has made it possible to execute our algorithm implementation on several platforms: drones, Raspberry Pi, and Android devices. In addition, the algorithm proposed here is not custom to a specific sensor or device; thus, the implementation can be adjusted to other mobile platforms. Experiments conducted using Samsung’s Galaxy S9 camera and the DJI’s Mavic Pro drone camera demonstrated that the image distortion is negligible and the star detection algorithm produces accurate results even without the need to calibrate the camera first. The algorithm proposed in this work copes with a certain level of lens distortion (as explained above) and relies on the sensor’s ability to detect starlight automatically. Our experiment shows that this can be achieved in some of the new mobile devices’ cameras; see [36]. The state-of-the-art algorithm Tetra [21], for the lost-in-space problem star-tracking problem, presents a fast identification algorithm (0.14 s per image) with a high success rate (≈95%). Although this algorithm has better run-time and precision results, it is not applicable for earth-based star frames since it was designed for space applications and a narrow fixed FOV.

**Figure 18:**
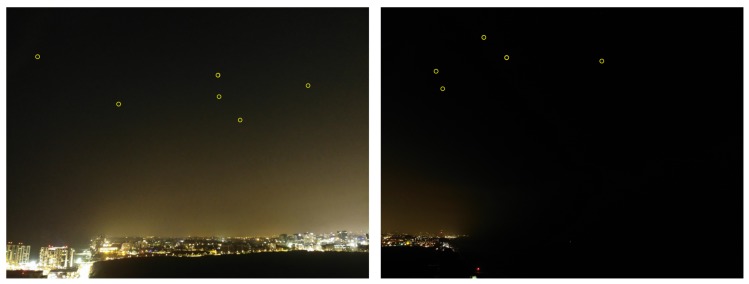
Star detection frames taken from a Mavic Pro drone. The stars can clearly be identified from these video-frames. The star tracking algorithm can serve as another sensor for detecting the drone orientation (especially when the GPS signal is lost).

**Figure 19:**
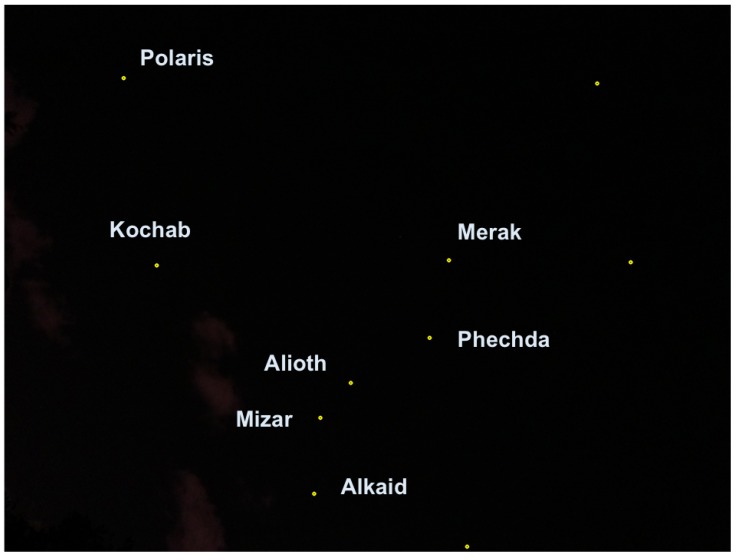
Star detection frame taken with a Raspberry Pi standard camera: Sony IMX-219, auto-exposure of 1 second and IS0 160.

Modern camera-drones often have stabilized cameras (gimbal based) and high-end smart-phone grade sensors with improved lenses. Therefore, their camera is suitable for performing star tracking in flight. This may lead to the possibility of performing an “in-air” compass calibration, which is an essential mission-critical application. The authors found the following drones suitable for detecting stars in real-time (30 Hz) video: DJI’s Mavic Pro, Mavic 2.0 (Zoom and Pro), and Phantom 4 (Advanced and Pro); see Figure 18. In fact, most modern action cameras (such as GoPro5.0 and above) are suitable for detecting stars. As shown in the above section, even a low-cost Raspberry Pi camera (V2.1) is sensitive enough for detecting stars (see Figure 19) in a 1–10 Hz rate video—allowing even low-cost autonomous robotics to be able to compute their star-based global orientation in real time.

## 6. Conclusions

This work presented a novel star-tracker framework for mobile applications. The suggested new algorithm can report robust orientation even in sub-optimal conditions such as light pollution, partially cloudy sky, and outliers (such as airplanes). The rapid hashing process makes the algorithm suited for embedded low-cost devices. The proposed framework has several environmental limitations, including: massive light pollution, cloudy weather, building blockage and, naturally, a night time constraint. Thus, it can hardly be used for autonomous vehicles in an urban transportation system. However, the proposed star-tracker algorithm can be used by commercial micro drones in proper star-visibility conditions. It should be noted that, in daytime conditions, the orientation can be approximated using the position of the sun (and the time and location), see: [37]. Based on field experiments, we conclude that modern smartphones are capable of detecting stars and computing the expected orientation even in sub-optimal scenarios. Combined with MEMS gyro, the star-tracker orientation can be reported in high rates. The use of such accurate orientation sensors may benefit a wide range of mobile platforms, including autonomous vessels, UAV drones, and nano-satellites. Figure 18 shows the feasibility of activating the star tracking algorithm on a COST drone camera. Figure 16 demonstrates a typical orientation accuracy with [0.1°,0.3°] angular error, which is significantly better than existing COTS orientation sensors. We expect that, with a better lens manufacturing, the expected accuracy of the suggested algorithms can be further improved and reach the “pixel” level of accuracy, which is commonly [0.01°,0.05°] in recent cameras of mobile phones and drones. The presented algorithm is currently being implemented on a nano-satellite which is scheduled to be launched in Q4,2020; the satellite will be using a Raspberry-Pi Zero and a standard 8 mega-pixel Sony’s IMX219 camera for both imaging and star tracing applications.

## Figures and Tables

**Table 1:** Simulation runtime table.

AC	Stars	Triangles	Runtime (ms)
0.1	15	18.5	16.7
0.1	20	23.8	28.11
0.05	25	228.3	41.65
0.05	20	250	37.85
0.01	18	1007	341
0.01	21	1393	408.72
0.01	25	2388	740.75
